# Survival Analysis in Male Breast Cancer With Bone Metastasis Based on the SEER Database

**DOI:** 10.3389/fonc.2022.659812

**Published:** 2022-04-13

**Authors:** Xingjuan Zhou, Junwei Zhang, Yunqing Wang, Zhenguo Cao

**Affiliations:** ^1^ Department of Anatomy, Xuzhou Medical University, Xuzhou, China; ^2^ Department of Orthopedics, Second Affiliated Hospital of Xuzhou Medical University, Xuzhou, China

**Keywords:** breast cancer, bone metastasis, clinicopathological characteristics, survival, risk factors

## Abstract

**Purpose:**

Breast cancer (BC) has been extensively and deeply studied as the number one malignant tumor in women, but its status in male patients, especially in male metastatic patients, is rarely reported. Thus, this study aimed to explore the prognosis and risk factors of male BC with bone metastasis.

**Patients and Methods:**

We searched the Surveillance, Epidemiology, and End Results (SEER) database to identify all patients diagnosed with male BC with bone metastasis from 2010 to 2016. Risk factors of overall survival (OS) and cancer-specific survival (CSS) were analyzed by univariable and multivariable Cox analyses. We also drew Kaplan–Meier plots to show the correlation between independent risk factors and survival.

**Results:**

A total of 207 male BC patients with bone metastasis were included for analysis. Approximately one-third of patients also had lung metastasis. Luminal A subtype comprised 58.5% of the overall patient population. These patients had a poor prognosis, with 3-year OS and CSS rates, 36.7% and 39.5%, respectively. Further analysis revealed that age ≤60 years old, luminal A or B, and surgery were independent predictors of prolonged OS and CSS. On Cox multivariable analysis, brain metastasis was associated with OS and not CSS.

**Conclusion:**

We identified four independent factors associated with prognosis in male BC patients with bone metastasis, namely age, tumor subtype, surgery, and brain metastasis. Knowing these risk factors will help clinicians make more appropriate treatment plans.

## Introduction

Male breast cancer (BC) is a rare malignancy representing less than 1% of all BCs and less than 1% of all male cancers (1, 2). With the increasing incidence of male BC in recent years (3, 4), researchers have begun to pay attention to the treatment and prognosis of this special group (5). At present, the treatment of male BC mainly refers to the treatment of female patients (6). Additionally, compared with female patients, male BC patients had a worse prognosis (7, 8). Bone is not only the most common metastatic site for female BC, but it is also the most common metastatic site for male BC (5). As far as we know, clinical studies on systematic prognosis analysis of male BC patients with bone metastasis are lacking. To date, the standardized treatment of male BC with bone metastasis has not been proven.

Many previous studies have shown that male breast cancer is not the same as female disease (9, 10). Recently, Xie et al. (5)reported that metastatic male BC patients had unique clinicopathological characteristics, which were different from nonmetastatic male BC patients. We cannot help wondering how the prognosis of male BC with bone metastasis and whether its risk factors are the same as those of female patients? Therefore, we applied the Surveillance, Epidemiology and End Results (SEER) database to solve the above questions, which is the largest population database for clinical cancer research. Our findings may provide a better understanding of, male BC with bone metastasis and further improve their prognosis.

## Material and Methods

### Study Population

Clinical data on BC with bone metastasis were retrieved by using the SEER*Stat version 8.3.8. Since the database only included patients diagnosed with bone metastases after 2010, we only included patients from 2010 to 2016. This population-based database collects information on cancer patients in 18 registries, representing nearly 30% of the US population (www.seer.cancer.gov). In the current study, we included clinicopathological data, sociological data, and treatment data. This study obtained approval from our institutional review board.

**Figure 1:**
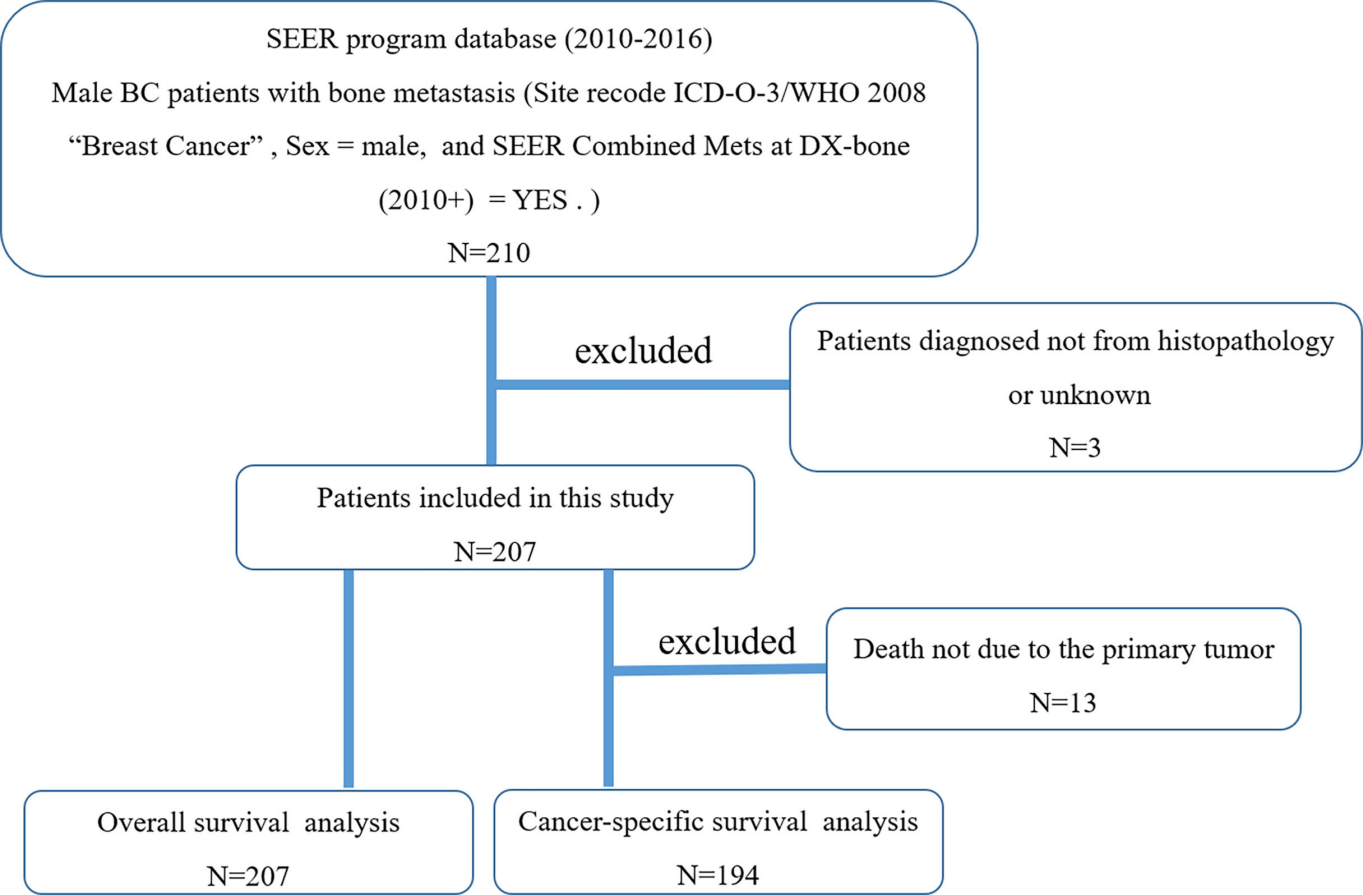
The flowchart for selection of study population. (SEER, Surveillance, Epidemiology, and End Results; ICD-O-3, International Classification of Diseases for Oncology, 3rd edition; BC, breast cancer).

When selecting target patients, we define three keywords, namely male, breast cancer, and bone metastasis. Cases without histopathological diagnosis were excluded (*n* = 3). The patient selection flowchart is shown in **Figure 1**. Surgery or radiotherapy in this study refers to the primary BC (11). Based on previous literature (12, 13), CSS is defined as the time from initial diagnosis to death due to BC itself. All patients were initially diagnosed with breast cancer and bone metastasis (stage IV), and follow-up surgery refers to surgery on the primary site.

### Statistical Methods

We first performed the univariable Cox regression analyses to rule out nonsignificant survival predictors. We then included statistically significant factors into multivariate Cox regression analysis to identify independent risk factors. At the same time, we calculated hazard ratios (HRs) and 95% confidence interval (CI). We drew survival curves to show the relationship between independent risk factors and survival and applied the log-rank test method for comparative analysis. Variables with two-tailed *p* < 0.05 were considered statistically significant. All statistical analyses were performed by using IBM SPSS Statistics 21.

## Results

### Patient Characteristics

**Table 1** summarizes the baseline characteristics of 207 male BC patients with bone metastasis identified from the SEER database. Of 207 patients, 74.9% were white. More than half of the patients were aged over 60 years old. High tumor grade was detected in 39.1% of cases. The pathological type of most patients (*n* = 170, 82.1%) was ductal and lobular neoplasms. In total, 58.5% of cases presented luminal A, 17.9% presented luminal B, and 9.2% presented triple negative. Tumor size distribution was 55.6% and 30.0% for <5 and ≥5 cm, respectively. Distant organ metastasis included the lung (35.7%), liver (13.0%), and brain (8.7%). More than three-quarters (78.7%) of the patients were insured. Over half of the patients were married. In terms of treatment-related variables, 67 (32.4%) patients received surgery, 77 (37.2%) received radiotherapy, and 99 (47.8%) received chemotherapy. Three-year OS and CSS rates for all cases were 36.7% and 39.5%, respectively.

**Table 1:** Baseline characteristics of 207 male breast cancer with bone metastasis.

Variable	Value
**Race**	
White	155 (74.9%)
Black	39 (18.8%)
Others	13 (6.3%)
**Age (years)**	
≤60	82 (39.6%)
>60	125 (60.4%)
Mean	64
Median	65
**Tumor grade**	
Low grade	84 (40.6%)
High grade	81 (39.1%)
Unknown	42 (20.3%)
**Histologic subtype**	
Ductal and lobular neoplasms	170 (82.1%)
Others	37 (17.9%)
**Tumor subtype**	
Luminal A	121 (58.5%)
Luminal B	37 (17.9%)
Triple negative	19 (9.2%)
Unknown	30 (14.5%)
**Tumor size (cm)**	
<5	115 (55.6%)
≥5	62 (30.0%)
Unknown	30 (14.5%)
**Surgery**	
Yes	67 (32.4%)
No	140 (67.6%)
**Radiotherapy**	
Yes	77 (37.2%)
No	130 (62.8%)
**Chemotherapy**	
Yes	99 (47.8%)
No	108 (52.2%)
**Brain metastasis**	
No	189 (91.3%)
Yes	18 (8.7%)
**Liver metastasis**	
No	180 (87.0%)
Yes	27 (13.0%)
**Lung metastasis**	
No	133 (64.3%)
Yes	74 (35.7%)
**Insurance status**	
Insured	163 (78.7%)
Others	40 (19.3%)
Unknown	4 (1.9%)
**Marital status**	
Married	108 (52.2%)
Others	87 (42.0%)
Unknown	12 (5.8%)
**Dead**	
Yes	116 (56.0%)
No	91 (44.0%)
**1-Year OS rate**	69.70%
**1-Year CSS rate**	70.30%
**3-Year OS rate**	36.70%
**3-Year CSS rate**	39.50%

Low grade: ICD-O-3 grade 1 (well-differentiated) and grade 2 (moderately differentiated). High grade: ICD-O-3 grade 3 (poorly differentiated) and grade 4 (undifferentiated anaplastic). OS, overall survival; CSS, cancer-specific survival.

### Survival Analysis

On univariable analysis, variables found to be significantly associated with OS and CSS were age, histologic subtype, tumor subtype, surgery, brain metastasis, and liver metastasis (**Table 2**). There was no significant difference in OS or CSS by race, tumor grade, tumor size, radiotherapy, chemotherapy, lung metastasis, insurance status, and marital status (**Table 2**).

**Table 2:** Univariate Cox analysis of variables in male breast cancer with bone metastasis.

Variable	OS		CSS	
	HR (95% CI)	*p*	HR (95% CI)	*p*
**Race**				
White	1		1	
Black	1.066 (0.670–1.696)	0.788	1.003 (0.605–1.662)	0.991
Others	1.376 (0.634–2.988)	0.42	1.429 (0.620–3.293)	0.402
**Age (years)**				
≤60	1		1	
>60	1.667 (1.121–2.477)	**0.012**	1.762 (1.153–2.691)	**0.009**
**Tumor grade**				
Low grade	1		1	
High grade	0.918 (0.598–1.409)	0.695	0.991 (0.623–1.577)	0.97
**Histologic subtype**				
Ductal and lobular neoplasms	1		1	
Others	2.500 (1.614–3.872)	**<0.001**	2.557 (1.629–4.014)	**<0.001**
**Tumor subtype**				
Luminal A	1		1	
Luminal B	0.866 (0.508–1.474)	0.595	0.862 (0.488–1.521)	0.607
Triple negative	4.857 (2.802–8.419)	**<0.001**	4.777 (2.701–8.448)	**<0.001**
**Tumor size (cm)**				
<5	1		1	
≥5	1.475 (0.981–2.218)	0.062	1.368 (0.882–2.124)	0.162
**Surgery**				
Yes	1		1	
No	2.180 (1.437–3.306)	**<0.001**	2.154 (1.382–3.357)	**0.001**
**Radiotherapy**				
Yes	1		1	
No	1.156 (0.794–1.682)	0.45	1.150 (0.770–1.718)	0.494
**Chemotherapy**				
Yes	1		1	
No	1.171 (0.810–1.691)	0.401	1.211 (0.820–1.790)	0.336
**Brain metastasis**				
No	1		1	
Yes	2.614 (1.448–4.719)	**0.001**	2.426 (1.282–4.588)	**0.006**
**Liver metastasis**				
No	1		1	
Yes	1.906 (1.172–3.099)	**0.009**	1.894 (1.146–3.128)	**0.013**
**Lung metastasis**				
No	1		1	
Yes	1.203 (0.829–1.747)	0.33	1.207 (0.811–1.795)	0.354
**Insurance status**				
Insured	1		1	
Others	0.895 (0.562–1.428)	0.642	0.858 (0.520–1.416)	0.549
**Marital status**				
Married	1		1	
Others	1.192 (0.815–1.745)	0.366	1.094 (0.729–1.641)	0.665

Variables with bold values were statistically significant.

**Figure 2:**
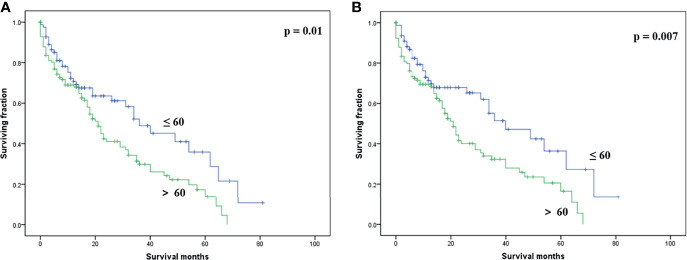
Kaplan–Meier method-estimated OS **(A)** and CSS **(B)** male breast cancer with bone metastasis stratified by age. (OS, overall survival; CSS, cancer-specific survival).

**Figure 3:**
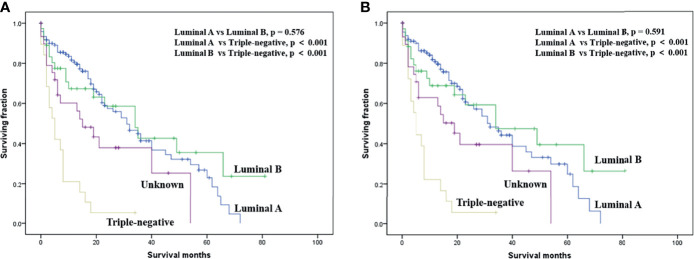
Kaplan–Meier method-estimated OS **(A)** and CSS **(B)** male breast cancer with bone metastasis stratified by tumor subtype. (OS, overall survival; CSS, cancer-specific survival).

**Figure 4:**
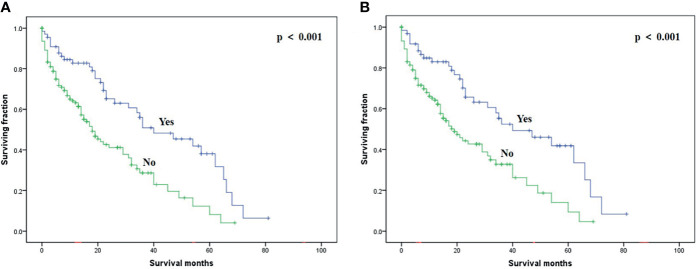
Kaplan–Meier method-estimated OS **(A)** and CSS **(B)** male breast cancer with bone metastasis stratified by surgery. (OS, overall survival; CSS, cancer-specific survival).

**Figure 5:**
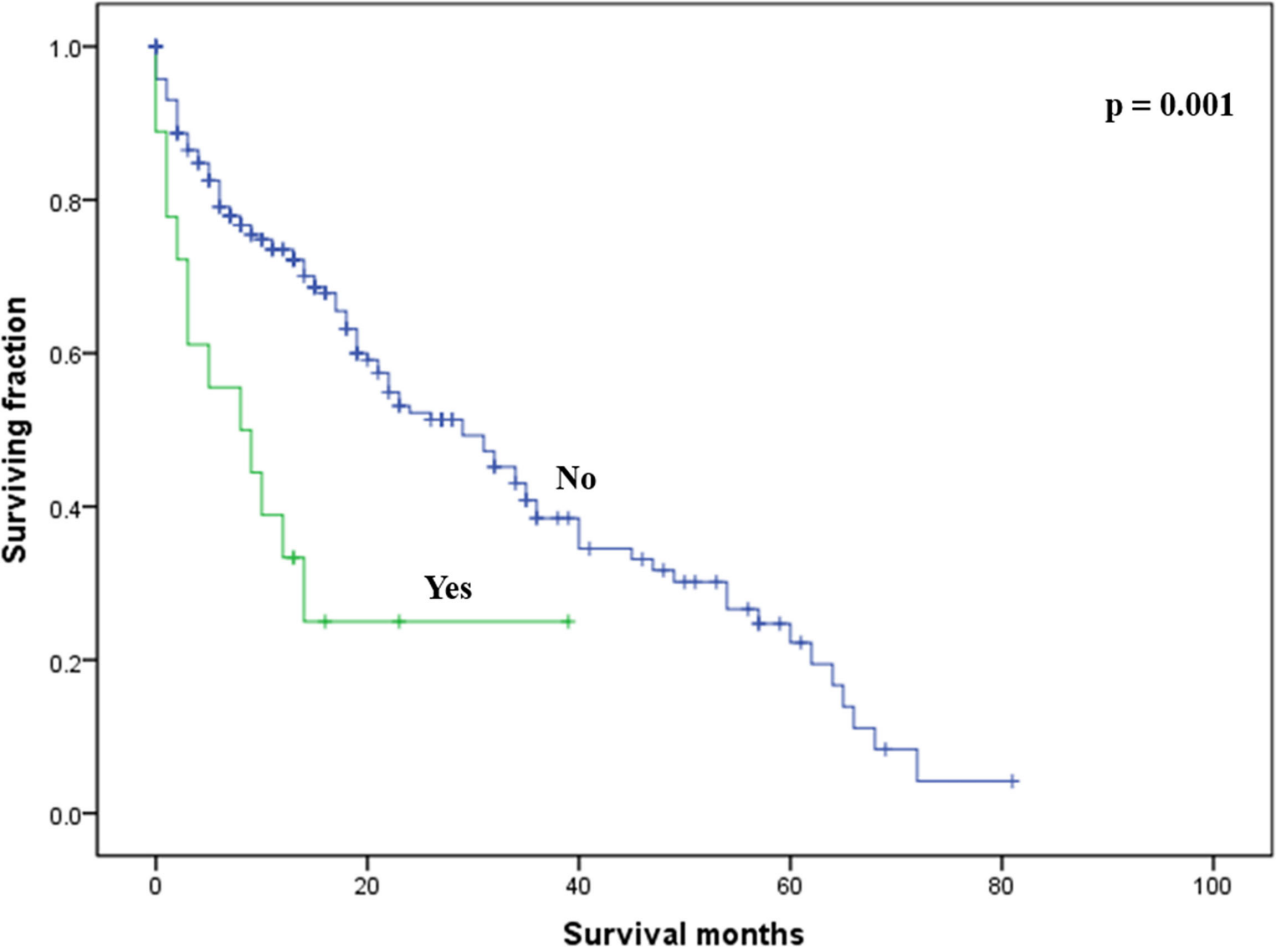
Kaplan–Meier method-estimated OS male breast cancer with bone metastasis stratified by brain metastasis. (OS, overall survival).

On multivariable analysis, age over 60 years old, other histologic subtypes, triple-negative subtype, no surgery, and brain metastasis were independent predictors of decreased OS (**Table 3**). Multivariable analysis revealed age, histologic subtype, tumor subtype, and surgery were significant predictors for CSS (**Table 3**). The Kaplan–Meier survival curves showed that patients with age ≤60 years old (**Figure 2**), luminal A or B (**Figure 3**), or surgery (**Figure 4**) had better OS and CSS. Moreover, brain metastasis had a negative influence on OS (**Figure 5**) but not CSS.

**Table 3:** Multivariate Cox analysis of variables in male breast cancer with bone metastasis.

Variable	OS		CSS	
	HR (95% CI)	*p*	HR (95% CI)	*p*
**Age (years)**				
≤60	1		1	
>60	1.671 (1.110–2.515)	**0.014**	1.806 (1.159–2.815)	**0.009**
**Histologic subtype**				
Ductal and lobular neoplasms	1		1	
Others	1.205 (0.674–2.155)	0.53	1.236 (0.678–2.255)	0.489
**Tumor subtype**				
Luminal A	1		1	
Luminal B	0.881 (0.507–1.530)	0.652	0.955 (0.526–1.734)	0.881
Triple negative	3.029 (1.455–6.303)	**0.003**	3.025 (1.427–6.412)	**0.004**
**Surgery**				
Yes	1		1	
No	1.764 (1.132–2.749)	**0.012**	1.734 (1.080–2.784)	**0.023**
**Brain metastasis**				
No	1		1	
Yes	2.045 (1.082–3.865)	**0.028**	1.950 (0.982–3.872)	0.056
**Liver metastasis**				
No	1		1	
Yes	1.293 (0.744–2.248)	0.362	1.330 (0.755–2.341)	0.324

Variables with bold values were statistically significant.

## Discussion

With the popularization of precision medicine, it is necessary to discuss the clinical difficulty of male BC with bone metastasis. This study first explored the factors associated with prognosis in BC patients with bone metastasis based on the public SEER database. This study found that the significant independent predictors affecting BC with bone metastasis were not as many as expected, including age, tumor subtype, surgery, and brain metastasis. The results of this study provide an important reference value for clinicians to guide patients to receive personalized treatment. In addition, this study is also a good start for clinical research on male BC with bone metastasis.

On the whole, the prognosis of male BC with bone metastasis (3-year OS and CSS rates: 36.7% and 39.5%) was worse than that of female patients (3-year OS and CSS rates: 51.7% and 53.6%) (13), suggesting that the prognosis and treatments of such patients need more attention. Previous studies indicated that older BC patients were prone to bone metastasis (14) and age was an important independent predictor of survival (15, 16). Our multivariable results also highlighted this finding in male BC patients with bone metastasis. A significant difference in survival was not revealed among various races, which was congruent with some previous studies (13, 17). However, other studies found race was an independent prognostic factor among BC with bone metastasis (15, 16). Tumor grade is usually recognized as an independent risk factor for the prognosis of BC (16, 18). Wang et al. (13) recently identified higher tumor grade was an independent predictor of worse survival among female BC patients with bone metastasis. However, this study failed to identify tumor grade as a significant risk factor for survival.

Several researchers have reported an effect of histologic subtype on survival among BC with bone metastasis (13, 19). Although the univariable analysis suggested that the histologic subtype was a significant risk factor affecting survival among our patients, the multivariable analysis did not support this finding. The tumor subtype might be one of the most useful survival predictors in male BC patients with bone metastasis. In line with our traditional knowledge of breast cancer, those with a triple-negative subtype had the worst prognosis. In contrast to a prior study on female BC with bone metastasis (13), we noted that tumor size in the current study was not correlated with survival. Of note, the presence of brain metastasis was an independent risk factor associated with a decreased OS, not CSS. Lung or liver metastases seem to have little effect on prognosis in male BC patients with bone metastasis. Therefore, treatment of brain metastasis may have survival benefits in such patients. Additionally, insurance status and marital status had no association with survival in this study.

At present, standard treatments of BC with bone metastasis have not been established, let alone the treatments of male BC with bone metastasis. In our study, surgery of primary sites was an effective treatment method to prolong the prognosis of male BC with bone metastasis, which was consistent with the situation of female BC patients with bone metastasis (13, 17). Wang et al. (13) found that chemotherapy can significantly improve the prognosis of female BC with bone metastasis, while radiotherapy has no significant effect on prognosis. Interestingly, chemotherapy and radiotherapy did not improve the prognosis of male BC with bone metastasis. Further validation of the different treatment methods of such patients is clinically required.

We need to point out some limitations presented in this study. First, the retrospective nature of this study can lead to bias. Second, endocrine therapy information is not available in the database. Third, recurrence or metastasis data during follow-up were also not available in the database. Additionally, the sample size of this study was relatively small. Relevant clinical studies with larger sample sizes can be carried out in the future.

## Conclusions

This is the largest study of survival analysis on male BC patients with bone metastasis. Age, tumor subtype, surgery, and brain metastasis were identified as independent risk factors of survival. Surgery of the primary tumors is recommended for such populations. However, more studies are needed to confirm our results and identify more survival predictors in the future.

## Data Availability Statement

The raw data supporting the conclusions of this article will be made available by the authors, without undue reservation.

## Ethics Statement

The studies involving human participants were reviewed and approved by the Institutional Review Board of Xuzhou Medical University. Written informed consent for participation was not required for this study in accordance with the national legislation and the institutional requirements.

## Author Contributions

XZ and ZC conceived and designed the study. XZ and JZ collected the data. XZ, JZ and YW performed the statistical analysis. XZ wrote the manuscript and ZC revised it. All authors read and approved the final manuscript.

## Conflict of Interest

The authors declare that the research was conducted in the absence of any commercial or financial relationships that could be construed as a potential conflict of interest.

## Publisher’s Note

All claims expressed in this article are solely those of the authors and do not necessarily represent those of their affiliated organizations, or those of the publisher, the editors and the reviewers. Any product that may be evaluated in this article, or claim that may be made by its manufacturer, is not guaranteed or endorsed by the publisher.

## References

[1] Korde LA Zujewski JA Kamin L Giordano S Domchek S Anderson WF. Multidisciplinary Meeting on Male Breast Cancer: Summary and Research Recommendations. J Clin Oncol Off J Am Soc Clin Oncol (2010) 28(12):2114–22. doi: 10.1200/jco.2009.25.5729 PMC2860409 20308661

[2] Weiss JR Moysich KB Swede H. Epidemiology of Male Breast Cancer. Cancer Epidemiol Biomarkers Prev Publ Am Assoc Cancer Research cosponsored by Am Soc Prev Oncol (2005) 14(1):20–6. 15668471

[3] Speirs V Shaaban AM. The Rising Incidence of Male Breast Cancer. Breast Cancer Res Treat (2009) 115(2):429–30. doi: 10.1007/s10549-008-0053-y 18478326

[4] Reddington R Galer M Hagedorn A Liu P Barrack S Husain E. Incidence of Male Breast Cancer in Scotland Over a Twenty-Five-Year Period (1992-2017). Eur J Surg Oncol J Eur Soc Surg Oncol Br Assoc Surg Oncol (2020) 46(8):1546–50. doi: 10.1016/j.ejso.2020.01.009 31955992

[5] Xie J Ying YY Xu B Li Y Zhang X Li C. Metastasis Pattern and Prognosis of Male Breast Cancer Patients in US: A Population-Based Study From SEER Database. Ther Adv Med Oncol (2019) 11:1758835919889003. doi: 10.1177/1758835919889003 31798694 PMC6859799

[6] Gómez-Raposo C Zambrana Tévar F Sereno Moyano M López Gómez M Casado E. Male Breast Cancer. Cancer Treat Rev (2010) 36(6):451–7. doi: 10.1016/j.ctrv.2010.02.002 20193984

[7] Liu N Johnson KJ Ma CX. Male Breast Cancer: An Updated Surveillance, Epidemiology, and End Results Data Analysis. Clin Breast Cancer (2018) 18(5):e997–e1002. doi: 10.1016/j.clbc.2018.06.013 30007834

[8] Wang Y Chen K Yang Y Tan L Chen L Zhu L. Incidence and Survival Outcomes of Early Male Breast Cancer: A Population-Based Comparison With Early Female Breast Cancer. Ann Trans Med (2019) 7(20):536. doi: 10.21037/atm.2019.10.04 PMC6861739 31807518

[9] Fentiman IS. Male Breast Cancer Is Not Congruent With the Female Disease. Crit Rev Oncol/Hematol (2016) 101:119–24. doi: 10.1016/j.critrevonc.2016.02.017 26989051

[10] Gucalp A Traina TA Eisner JR Parker JS Selitsky SR Park BH. Male Breast Cancer: A Disease Distinct From Female Breast Cancer. Breast Cancer Res Treat (2019) 173(1):37–48. doi: 10.1007/s10549-018-4921-9 30267249 PMC7513797

[11] Wang Z Chen G Chen X Huang X Liu M Pan W. Predictors of the Survival of Patients With Chondrosarcoma of Bone and Metastatic Disease at Diagnosis. J Cancer (2019) 10(11):2457–63. doi: 10.7150/jca.30388 PMC6584356 31258751

[12] Wang Z Wu B Zhou Y Huang X Pan W Liu M. Predictors of the Survival of Primary and Secondary Older Osteosarcoma Patients. J Cancer (2019) 10(19):4614–22. doi: 10.7150/jca.32627 PMC6746122 31528225

[13] Wang Z Cheng Y Chen S Shao H Chen X Wang Z. Novel Prognostic Nomograms for Female Patients With Breast Cancer and Bone Metastasis at Presentation. Ann Trans Med (2020) 8(5):197. doi: 10.21037/atm.2020.01.37 PMC7154431 32309344

[14] Diessner J Wischnewsky M Stüber T Stein R Krockenberger M Häusler S. Evaluation of Clinical Parameters Influencing the Development of Bone Metastasis in Breast Cancer. BMC Cancer (2016) 16:307. doi: 10.1186/s12885-016-2345-7 27175930 PMC4865990

[15] Tu Q Hu C Zhang H Peng C Kong M Song M. Establishment and Validation of Novel Clinical Prognosis Nomograms for Luminal A Breast Cancer Patients With Bone Metastasis. BioMed Res Int (2020) 2020:1972064. doi: 10.1155/2020/1972064 33490234 PMC7787749

[16] Li X Zhang X Liu J Shen Y. Prognostic Factors and Survival According to Tumour Subtype in Women Presenting With Breast Cancer Bone Metastases at Initial Diagnosis: A SEER-Based Study. BMC Cancer (2020) 20(1):1102. doi: 10.1186/s12885-020-07593-8 33187507 PMC7666499

[17] Liu D Wu J Lin C Andriani L Ding S Shen K. Breast Subtypes and Prognosis of Breast Cancer Patients With Initial Bone Metastasis: A Population-Based Study. Front Oncol (2020) 10:580112. doi: 10.3389/fonc.2020.580112 33344236 PMC7739957

[18] Huang Z Hu C Liu K Yuan L Li Y Zhao C. Risk Factors, Prognostic Factors, and Nomograms for Bone Metastasis in Patients With Newly Diagnosed Infiltrating Duct Carcinoma of the Breast: A Population-Based Study. BMC Cancer (2020) 20(1):1145. doi: 10.1186/s12885-020-07635-1 33238981 PMC7687803

[19] Lopez-Tarruella S Escudero MJ Pollan M Martín M Jara C Bermejo B. Survival Impact of Primary Tumor Resection in *De Novo* Metastatic Breast Cancer Patients (GEICAM/El Alamo Registry). Sci Rep (2019) 9(1):20081. doi: 10.1038/s41598-019-55765-9 31882586 PMC6934456

